# Cessation outcomes in adult dual users of e-cigarettes and cigarettes: The Population Assessment of Tobacco and Health cohort study, United States (2013–2016)

**DOI:** 10.1007/s00038-020-01436-w

**Published:** 2020-07-24

**Authors:** Olatokunbo Osibogun, Zoran Bursac, Martin Mckee, Tan Li, Wasim Maziak

**Affiliations:** 1 Department of Epidemiology, Robert Stempel College of Public Health & Social Work, Florida International University, Miami, FL, USA; 2 Department of Biostatistics, Robert Stempel College of Public Health & Social Work, Florida International University, Miami, FL, USA; 3 London School of Hygiene & Tropical Medicine, London, UK

**Keywords:** e-cigarettes, dual use, harm reduction, tobacco cessation, adults

## Abstract

**Objectives:**

We examined the transitions of adult dual e-cigarette/cigarette users in the United States (US) in relation to nicotine dependence (ND) symptoms, interest in quitting, and history of cardiovascular disease (CVD).

**Methods:**

We used the Population Assessment of Tobacco and Health study Waves 1 and 3 (2013–2016) in a longitudinal analysis of adults (≥18years). Dual past-month users of e-cigarettes/cigarettes were identified from Wave 1 and followed for tobacco use transitions two years later (Wave 3).

**Results:**

Among 1,870 adult dual users at Wave 1, 25.7% (95% CI 23.5–28.2) were dual users two years later, 12.1% (95% CI 10.6–13.7) reported no past-month tobacco use, 7.0% (95% CI 5.6–8.9) e-cigarette mono-use, and 55.2% (95% CI 52.4–58.0) cigarette mono-use. In the regression analysis, greater ND severity was associated with decreased risk of no past-month tobacco use (RRR 0.29; 95% CI 0.12–0.71). Interest in quitting and CVD factors were not associated with no past-month tobacco or e-cigarette mono-use.

**Conclusion:**

Dual users who are nicotine dependent are less likely to transition to cessation. To quit cigarette use, other cessation resources may be necessary to support the needs of cigarette smokers who use e-cigarettes, particularly those at risk of continuing cigarette smoking or those with smoking-related illness.

## Introduction

The spread of e-cigarette use in many countries has brought front and center the debate about their place in reducing the burden of tobacco-related disease (National Academies of Sciences, Engineering and Medicine; NASEM 2018). Two questions arise. The first is what will be the net impact on smoking rates? Thus, if as is claimed, (McNeill et al. 2018) that e-cigarettes might help adult smokers quit, is this counterbalanced by youth initiation? (Warner & Mendez 2018) The second and more fundamental question is whether e-cigarettes can contribute to tobacco harm reduction (THR). This is based on the premise that adult smokers who cannot do without nicotine may be able to reduce their risk by shifting from conventional cigarettes or quitting altogether (Abrams et al. 2018; Khoudigian et al., 2016; Andler et al., 2015). Indeed, evidence from a randomized controlled trial show that when accompanied by behavioral support e-cigarettes in health care setting were more effective for smoking cessation than nicotine-replacement therapy (Hajek et al. 2019).

From this perspective, advocating e-cigarettes to those suffering from smoking-related diseases but who are unable to quit, such as patients with cardiovascular disease (CVD), could be beneficial if it enables them to transition to sole e-cigarette use or, even better, ultimately to abstain from both. Yet outside of the confines of tightly controlled clinical trials, clinicians trying to help such individuals are currently in uncharted territory without any definitive evidence to support their decision-making (Brandon et al. 2015; Drummond and Upson 2014). Although, reports from the United States Preventive Services Task Force suggest that there is insufficient evidence to recommend e-cigarettes as a cessation tool (USPSTF 2015), clinicians also run the risk of exposing their patients to additional risk either by advising the use of a potentially ineffective or even hazardous (Alzahrani et al. 2018) cessation tool, or by withholding what could be a valuable means to help them reduce their harm (NASEM 2018). Additionally, with tobacco use identified as one of the modifiable risk factor for CVD (USDHHS 2014) and current research suggesting that e-cigarettes have acute adverse effects on the cardiovascular system (Bhatnagar 2016), it is important to examine tobacco use transitions in this subpopulation that is particularly vulnerable to the effects of tobacco use.

What is needed is research on the potential e-cigarettes might have to help adult smokers reduce their harm in a real-world population setting, using appropriate study design and extended follow up (Barrington-Trimis et al. 2015; Dutra and Glantz 2014). The 2018 report on e-cigarettes by the NASEM emphasized that because the e-cigarette phenomenon is relatively recent, “the majority of studies … lack sufficient duration of follow-up to study the naturalistic cigarette smoking progression sequence” (NASEM 2018).

In 2011, the NIH and FDA initiated the Population Assessment of Tobacco and Health study (PATH), as the first population cohort to study tobacco use since Congress gave FDA the authority to regulate tobacco products in 2009 (USDHHS 2020; Hyland et al. 2017). Studies from PATH Waves 1 and 2 began answering some important questions about e-cigarette transitions during the first year of follow-up (Coleman et al. 2018; Verplaeste et al. 2018). For example, Coleman and colleagues found that majority of adult dual users in Wave 1 (87.8%) either continued dual use or relapsed to cigarette-only smoking at one year follow up, compared to 12.1% who discontinued cigarette smoking or continued with e-cigarettes (Coleman et al. 2018). With the recent availability of Wave 3 data from PATH, we have the opportunity to look at an extended two-year follow-up of PATH’s data to examine a question that is central to the THR debate; can e-cigarettes help adult smokers who are unable to quit reduce their harm in a real-world setting? This allows us to answer the key question facing clinicians faced with patients who are heavy smokers who need but are unable to quit. Therefore, we investigated this question among adult dual e-cigarette/cigarette users who are nicotine dependent, interested in quitting, and have a clinical condition such as cardiovascular disease (CVD) that warrants their quitting, transition to sole e-cigarette use or total abstinence for an extended follow-up period.

## Methods

### Study Sample

The PATH Study is a collaboration between the NIH and FDA to inform the FDA’s regulatory approach to different tobacco products in the US (USDHHS 2020). PATH is an annual, nationally representative longitudinal study of persons 12 years and older who are residents of households, and noninstitutionalized civilians. Details have been described elsewhere (Hyland et al. 2017). The weighted response rate for household screening was 54%. Subsequently, following household screening, the response rate was 74.0% at Wave 1, yielding 32,320 participants, 83.2% at Wave 2, yielding 28,362 participants and 78.4% at Wave 3 yielding 28,148 participants. We examined tobacco use among adults who reported past-month (past 30 days) dual use of e-cigarette/cigarette at Wave 1 (2013–2014) and had follow-up information at Wave 3 (2015–2016). We examined three main transitions (from Waves 1–3) among these dual users (cessation, harm-reduction, cigarette transition), seeking their associations with the following characteristics at Wave 1; 1) ND symptoms, 2) interest in quitting, and 3) clinical CVD factors, in addition to baseline socioeconomic and behavioral factors (Cohn et al. 2015). The Institutional Review Board of Florida International University reviewed the study and deemed it exempt.

### Study Measures

#### Assessment of tobacco use

The PATH Study enquires about several tobacco products including cigarettes, e-cigarettes, cigars, cigarillos, little-filtered cigar, hookah, and smokeless tobacco (USDHHS 2020). Since combined e-cigarette/cigarette use is currently the most common and important e-cigarette use pattern (Owusu et al. 2019) from a policy and regulatory standpoint, we defined *past-month dual users* as those who reported past 30 days use of e-cigarettes and cigarettes in Wave 1, regardless of other tobacco products used. We followed these dual users 2 years later, defining three main trajectories based on their transitioning to; 1) no past-month use of any tobacco/nicotine product (*cessation transition*); 2) past-month mono e-cigarette use *(harm reduction transition*); or 3) past-month mono cigarette smoking *(cigarette transition)* at Wave 3. Only participants with complete information on dual use were used in this study (n=1,870).

#### Demographic and behavioral factors

Demographic variables included age, sex, sexual orientation, race/ethnicity, education, income, employment status, and census region. Age, sexual orientation, race and ethnicity, education, employment, census region and BMI were categorized as shown in Table 1 (USDHHS 2020; Cohn et al. 2015).

Behavioral factors assessed in Wave 1 included age at first exposure to a tobacco product, duration of tobacco product use, other tobacco use, marijuana use and alcohol consumption. Age at first exposure to tobacco products was categorized into <18, and ≥18 years. Duration of tobacco use was derived by subtracting the age at first exposure to tobacco product from participant’s age at Wave 1 and included as a continuous variable. Alcohol consumption and marijuana use were assessed from the questions pertaining to past-month drinking and marijuana use categorized into “yes” and “no”.

#### Nicotine dependence (ND) symptoms

*ND* assessment was based on several questions asked at Wave 1 for each tobacco product reported by participants. Since PATH did not include full scales for ND, but rather a selection of items from different scales, (Liu et al. 2017; DiFranza et al. 2002; Heatherthon et al. 1991) we opted for items that cover major domains of ND (e.g. craving, withdrawal, latency to smoke upon awakening, smoking heaviness), (Baker et al. 2013) simple to use in our modeling, and have been shown repeatedly to yield good measurement of ND (Baker et al. 2007). These were 1) time to first [product] use after waking up?; 2) do you consider yourself addicted to [product]?; 3) do you ever have strong cravings to smoke or use [product]?; 4) in the past 12 months, did you find it difficult to keep from smoking or using [product] in places where it was prohibited?; 5) have you ever felt like you really needed to use a [product]?; 6) did you cut down on activities that were enjoyable or important to you because [product] was not permitted at the activity?; and 7) frequency of tobacco use among past-month dual users categorized into everyday (regular), vs. some-day (not regular) use for both cigarette and e-cigarette (USDHHS 2020). All ND variables were dichotomized into binary scoring with “0” indicating “no” and “1” indicating “yes” as shown in Table 2. From these variables, we created a cumulative ND severity variable based on the number of endorsements of the 7 ND items used (range 0–7).

#### Interest in quitting

We used two items from PATH to assess interest in quitting. The first was interest in quitting (scale of 1–10; with 1, being not interested in quitting and 10, being extremely interested). We categorized this variable into tertiles: 1–3, 4–7 and 8–10. The second was past year quit attempts, measured as the “number of times tried to quit smoking/using tobacco product(s) in the past 12 months.” This was categorized into 0 (no quit attempt), and ≥1 (one or more quit attempts) (USDHHS 2020).

#### History of CVD-related illness

As our aim was to have an example of the potential effect of tobacco-associated clinical conditions on e-cigarette related transitions, we picked reports of the history of CVD and related conditions that are especially relevant to tobacco cessation and harm reduction. They were based on self-reported positive response to the questions asking, “Has a doctor, nurse or other health professionals ever told you that you have….” for diabetes mellitus (DM), heart attack (myocardial infarction, MI), high blood pressure and high cholesterol (yes, no) (USDHHS 2020). These CVD factors were assessed from participants at Wave 1 of the PATH study.

### Statistical analyses

We calculated descriptive statistics for the main transitions of dual tobacco use between Waves 1 and 3 (cessation, harm reduction, and cigarette transition). An outcome variable was derived to indicate respondents’ tobacco use transition at Wave 3 (0 = continued dual use, 1 = cessation (no tobacco use), 2 = harm reduction (mono e-cigarette use), 3 = cigarette transition (mono cigarette use).

The replicate weights provided by the PATH Study were used to obtain unbiased point and variance estimates using Fay’s Method of Balanced Repeated Replication, with the Fay coefficient value of 0.3, as recommended by the PATH Study team (USDHHS 2020). Weighted percentages for demographic and behavioral factors, ND symptoms, interest in quitting, and clinical CVD factors at Wave 1, were reported by with their corresponding 95% confidence intervals (CI) according to tobacco transitions at Wave 3. We used SVY procedures to report the p values for the differences among the tobacco use transitions and demographic and behavioral factors, ND symptoms, interest in quitting and clinical CVD factors respectively.

We applied multinomial logistic regression models to test the bivariate associations between the demographic, behavioral, ND, interest in quitting and clinical CVD factors at Wave 1 with the tobacco use transitions between Waves1 and 3. We reported unadjusted relative risk ratios (RRR) with their corresponding 95% CI for variables that had significant results only. For the multivariate predictors of main transitions among dual users (cessation, harm reduction, and cigarette transition) with continued dual use as reference, we fitted a multinomial regression model, adjusting for variables with inclusion p<0.2 from the bivariate analysis. Due to modest to moderate correlation and collinearity between individual variables within each domain (e.g. ND) we created dichotomous domain summary variables for ND symptoms [i.e. endorsing at least one symptom for the presence of an ND symptom (yes) versus the absence of symptoms (no)], interest in quitting (i.e. a score of 2 and above or reporting any quit attempt in the past year vs. score of 1 on quitting scale or no quit attempt) scoring and clinical CVD factors (i.e. responding positively to any clinical condition vs. no). These were categorized according to no endorsement of any ND, no interest in quitting/quit attempt, or no history of CVD factor as “no”, and any endorsement of any of domain components as “yes” for each domain.

Adjusted RRRs with their corresponding 95% CIs were calculated and reported. Finally, we tested the preplanned two-way interactions between the three domains of ND symptoms, interest in quitting, and history of CVD factors. The final analytic sample included 845 participants in the adjusted model following exclusion of participants with missing data on some of the assessed variables. Associations were considered statistically significant at the alpha level of 0.05. Analyses were performed using SVY procedures in STATA version 15·1 (StataCorp, College Station, TX).

## Results

### Transitions of dual use at two year follow up

Table 1 shows the descriptive statistics of dual use transitions between Waves 1 and 3 of PATH by main demographic and behavioral factors. Overall, 12.1% (95% CI 10.6–13.7) of dual users followed the cessation transition (i.e. to no past-month use of any tobacco product), 7.0% (95% CI 5.6–8.9) to the harm reduction transition (i.e. to mono e-cigarette use), 55.2% (95% CI 52.4–58.0) were in the cigarette transition (i.e., to mono cigarette use) and 25.7% (95% CI 23.3–28.2) remained as dual users at two-year follow up.

### Dual use transitions according to ND, interest in quitting, and clinical CVD factors

Overall, the majority of dual users reported at least one ND symptom (96.7%, 95% CI 95.9–97.4) and the most common symptom was tobacco use within 30 minutes (86.4%, 95% CI 84.3–88.1) and addiction to tobacco (86.2%, 95% CI 84.0–88.2) (Table 2). In terms of ND severity, 57.8% (95% CI 55.2–60.4) reported 4 to 5 ND symptoms. Over half of the participants (57.9%, 95% CI 54.8–60.9) had interest in quitting scores of 8–10, and 83.3% (95% CI 80.5–85.8) reported one or more quit attempts in the past year. Participants who reported at least one ND symptom were more likely to report transitioning to cigarette smoking (56.6%, 95% CI 53.4–59.7), while those who reported no ND symptoms were more likely to report cessation (49.8%, 95% CI 35.7–64.0) at 2-year follow up (*p<0.0001*). Dual users who reported an interest in quitting at Wave 1 were less likely to follow the cessation transition (10.7%, 95% CI 8.9–12.9) at Wave 3 and more likely to remain as dual users (28.0%, 95% CI 25.2–31.0) *(p=0.05)*. (Table 2)

A little over a third of participants (35.3%, 95% CI 32.4–38.3) overall reported a history of at least one clinical CVD-related factor at Wave 1. The distribution of the clinical conditions across the tobacco use transitions was not statistically significant but those who reported at least one condition were less likely to follow the cessation transition (9.2%, 95% CI 7.0–11.9) (Table 2).

### Predictors of cessation, harm reduction and cigarette transitions

In the bivariate analyses, among dual users at Wave 1, younger age at first tobacco exposure (0.46, 95% CI 0.29–0.73), and longer duration of tobacco use (0.98, 95% CI 0.96–0.99) were associated with decreased relative risks of cessation transition at Wave 3 (*p<0.05*; Table 4). Higher education (0.64, 95% CI 0.51–0.80) was associated with decreased relative risks of transitioning to cigarette smoking, while other tobacco use (1.64, 95% CI 1.18–2.29) and past-month marijuana use (1.74, 95% CI 1.14–2.65) were associated with increased relative risks of cessation transitions (Table 3).

The 3 main domains [ND symptoms (0.08, 95% CI 0.03–0.19), interest in quitting (0.26, 95% CI 0.10–0.74), and clinical conditions (0.59, 95% CI 0.40–0.88)], were associated with decreased relative risks of cessation compared to continuing dual use (Table 3). Similarly, participants who were in the higher categories of ND symptoms severity compared to 0–3 symptoms had a decreased relative risk of cessation (4–5 symptoms: 0.23, 95% CI 0.14–0.38; and 6–7 symptoms: 0.18, 95% CI 0.11–0.31) and harm reduction transitions (4–5 symptoms: 0.56, 95% CI 0.35–0.90; and 6–7 symptoms: 0.29, 95% CI 0.14–0.61) (Table 3).

In the final adjusted multivariate model, those who reported the age of first exposure to tobacco product of <18 years had a decreased relative risk of cessation transition (0.44, 95% CI 0.20–0.97) compared to ≥18 years. Also, ND severity of 4–5 symptoms (0.38, 95% CI 0.18–0.81) and 6–7 symptoms were associated with decreased relative risks of cessation (0.29, 95% CI 0.12–0.71) transition compared to dual use (Table 4). Interest in quitting and clinical CVD factors were not significantly associated with either cessation (1.30, 95% CI: 0.37–4.56 and 0.95, 95% CI: 0.44–2.05, respectively) or harm reduction (0.98, 95% CI: 0.08–11.27 and 0.75, 95% CI: 0.30–1.87, respectively) transitions. The interactions between ND symptoms, interest in quitting and clinical CVD factors domains were not statistically significant (*all p>0.05*).

## Discussion

These findings are important because dual e-cigarette/cigarette use has become the most common tobacco use pattern involving e-cigarettes in the US, with 55% of e-cigarette users also smoking (Sung et al. 2018). We have two main findings, each helping to inform the debate on the potential role of e-cigarettes in tobacco control.

The first is that among adult dual smokers, followed over 2 years in the PATH cohort study, 19.1% followed either cessation (12.1%) or harm reduction (7.0%) transitions, compared to 55.2% transitioning to cigarette use, and 25.7% continuing dual use. In other words, the majority transitioned to cigarette and less than one in five transitioned to a less harmful situation (i.e. to e-cigarettes or no tobacco use). Second, individuals who are highly addicted to nicotine were least likely either to quit or transition to a harm reduction scenario with the use of e-cigarettes.

These findings are important because they challenge certain widely aired views. One is that smokers taking up e-cigarettes are beginning a journey to reduced harm or even cessation (Glantz and Bareham 2018). However, the available evidence has often been marred by selectiveness of samples and outcomes, contradictory evidence, and mostly lacked length of follow-up to answer this question (NASEM 2018; Kalkhoran and Glantz 2016). There are many accounts of individuals who claim benefit in using e-cigarettes either to quit or reduce their harm by moving exclusively to e-cigarettes (Notley et al. 2018). Our findings confirm that such individuals exist. However, a majority of dual users transitioned to exclusive cigarette smoking, with a substantial minority remaining dual users. The latter is especially important given concerns that continued dual use may be associated with greater adverse health effects than with either on their own (Wang et al. 2018).

Another notion is that e-cigarettes may be especially useful to those who are highly addicted to nicotine, a group that includes many who have proven resistant to other interventions (Selva et al. 2018). Previous research suggests that dual users have greater dependence symptoms and cessation intentions than exclusive cigarette smokers (Rostron, Shroeder and Ambrose 2016), however one study found no impact of dual use on quit attempts (Etter and Eissenberg 2016). Additionally, another study found that nicotine dependence did not appear to moderate the use of e-cigarettes for reduction or cessation of cigarette smoking (Selya et al., 2018). E-cigarettes may be effective in smoking cessation for dependent smokers who seek help when combined with behavioral counselling compared to nicotine replacement therapy (Hajek et al., 2019). As a result, other cessation resources remain important in helping dependent smokers who want to quit (Babb et al., 2017).

Our findings are consistent with the few earlier, although smaller studies, with the closest in design being an Italian study that included 223 dual users followed over 2 years, and found that 14.3% followed the cessation transition, 12.5% the harm reduction transition, 16.6% continued dual use, and 57.4% transitioned to cigarettes (Manzoli et al. 2016). Another study from the US included 151 dual users among a larger sample of smokers but while 43.7% were still dual users at 2 years, the other data reported do not allow direct comparison with the present study (Zhuang et al. 2016).

These findings are of direct relevance to clinicians confronted with the dilemma of whether or not to advise their tobacco smoking patients who need to quit but cannot, to try e-cigarettes (Brandon et al. 2015). As noted, the notion that e-cigarettes can be the best option for those who could not or will not quit otherwise has been central in the THR debate (Abrams 2018; Warner 2018). While we did not find a statistically significant association between the CVD factors domain and cessation, probably due to low power to detect statistical significance, emerging evidence suggests that concurrent e-cigarette/cigarette use is associated with an increased risk of heart disease compared to each individually (Alzahrani et al. 2018). Additionally, studies suggest that the accessibility and availability of e-cigarettes may fuel nicotine addiction and promote dual use (Bhatnagar et al. 2016).

We observed a positive relationship between other tobacco use/marijuana use and cessation in the bivariate analysis. While some studies suggest that using other tobacco products during cessation could maintain nicotine levels following craving and withdrawal symptoms that make quitting difficult (USDHHS 2012; Dugas et al. 2020), others report that using other tobacco products may help lessen ND symptoms during cessation, allowing smokers adapt easily to the neurobiological changes seen with quitting (Dugas et al. 2020). Furthermore, similar to our study findings, previous studies suggest that marijuana use at baseline did not predict smoking cessation (Humfleet et al. 1999), a finding that is inconsistent with available research indicating a negative effect of marijuana use on cessation (Ford et al. 2002; Schauer et al. 2017).

## Strengths and limitations

This study has some limitations. As PATH did not include full scales for ND measurement, we limited our assessment of ND to a subset of available questions (USDHHS 2020). Previous research has demonstrated that individual items, several included here (e.g. time of first tobacco product; frequency of use), yield comparable measurements to the full scales (Baker et al. 2007). Second, although this analysis provides useful information on the transition from dual use over two time-points, we did not analyze participants’ behaviors between waves. However, our main aim was to track real-world evolution of dual use over an extended period of time rather than the dynamics of changes occurring during this period or factors influencing them. Third, tobacco use, interest in quitting and history of CVD were based on self-reports. Previous research, however, shows a good correlation between self-report of tobacco use and biomarkers of tobacco exposure, or clinical CVD and medical records (Yuji et al. 2004). Interest in quitting, moreover, was correlated with past year quit attempts (r= 0.20; *p<0.0001)*. Fourth, since this is a population-based study rather than a cessation trial, it was not possible to apply a definition of cessation based on prolonged abstinence as is used in intervention studies. Using such a definition would have led to even fewer people classified as the cessation or harm reduction transitions. Thus, if anything, our findings exaggerate the probability of cessation. Fifth, we had a number of missing data for some variables in our study, which may bias estimates, however, the PATH study involves weighting to account for nonresponse (Hyland et al., 2017; USDHHS 2020). Sixth, we were unable to assess the associations between the transition categories with ND symptoms, interest in quitting, and CVD clinical factors among only daily users of e-cigarettes in our study population. Due to small number of participants (n=106), some variables had relatively small cell sizes (sparse data) to achieve convergence in the regression models or to draw meaningful inferences. Finally, it is also possible that not all those with CVD or DM understood that smoking was detrimental to their health, and they may vary in their use of other cessation means (e.g. medications). While this concern is legitimate, it is unlikely that smokers with a history of MI for example would not be aware of how bad smoking is for their health. Also, the use of other cessation means should have led to moving away from the null effect we found. Notwithstanding, our study based on a representative sample of the US adult population, longitudinal design, extended follow up, and detailed use history of different tobacco products offer a unique opportunity to answer critical questions related to the role of e-cigarettes to help smokers quit or reduce their harm in a real-world setting.

## Conclusions

The present study shows that approximately one out of four dual users continue as dual users two years later. Nicotine dependence severity, and early age (<18 years) of 1^st^ exposure to tobacco were associated with decreased relative risks of following a cessation transition at 2-year follow up, while interest in quitting, and history of illness were not associated with favorable transitions towards cessation or harm reduction.

In order to quit cigarette use, other cessation resources may be important to support the needs of cigarette smokers who use e-cigarettes, particularly those at risk of continuing cigarette smoking or those with smoking-related illnesses. It seems that, with or without e-cigarettes, cessation and harm reduction success are very challenging in this population.

## Figures and Tables

**Table 1 –:** Prevalence characteristics of adult (≥18 years) dual e-cigarette/cigarette users according to main transitions at 2-year follow-up: Population Assessment of Tobacco and Health study, United States, 2013–2016

Wave 3	Overall %, (95% CI)	Cessation %, (95% CI)	Harm Reduction %, (95% CI)	Cigarette Transition %, (95% CI)	Dual use %, (95% CI)	P value
Wave 1						
***Total (n=1870)***	**100·0**	**12.1 (10.6–13.7)**	**7·0 (5·6–8·9)**	**55·2 (52·4–58·0)**	**25·7 (23·3–28·2)**	
**Demographic factors**						
***Age, years (n=1869)***						0.0011
18–24	17.7 (16.0–19.5)	20.0 (15.9–24.8)*	7.0 (4.7–10.4)	50.2 (44.3–56.1)*	22.8 (18·6–27·7)	
25–34	26·5 (24.0–29.2)	12.9 (9.7–16.9)	7.5 (4.9–11.1)	52.3 (46.9–57.6)	27.3 (22.8–32.5)	
≥35	55.8 (52.6–59.0)	9.2 (7.5–11.3)*	6.9 (5.0–9.4)	58.2 (54.4–62.0)*	25.7 (22.8–28.8)	
***Sex (n=1870)***						0.12
Male	47.6 (45.0–50.2)	13.6 (11.3–16.3)	7.9 (5.8–10.6)	53.3 (49.7–56.9)	25.2 (22.0–29.0)	
Female	52.4 (50.0–55.0)	10.6 (8.9–12.8)	6.3 (4.8–8.3)	57.0 (53.2–60.5)	26.1 (23.1–29.3)	
***Sexual orientation (n=1839)***						0.24
Heterosexual	91.4 (89.9–92.6)	12.0 (10.4–13.8)	6.8 (5.3–8.8)	55.9 (53.0–58.7)	25.3 (22.7–28.0)	
Lesbian/gay/bisexual/other	8.6 (7.4–10.1)	8.8 (5.5–13.9)	8.0 (4.6–13.6)	52·2 (44.6–59.6)	31.1 (25.0–38.0)	
***Race/Ethnicity***^a^ ***(n=1851)***						0.01
Non-Hispanic white	75.6 (73.4–77.7)	10.3 (8.8–12.0)*	7.3 (5.6–9.6)	55.0 (51.8–58.3)	27.4 (24.6–30.4)*	
Non-Hispanic black	7.5 (6.4–8.7)	14.8 (9.7–21.9)	6.9 (3.0–15.3)	61.1(51.3–70.0)	17.2 (11.0–26.0)*	
Hispanic	11.4 (9.9–13.2)	21.2 (15.2–28.9)*	5.4 (3.1–9.2)	51.1 (43.7–58.3)	22.3 (17.6–27.8)	
Other	5.5 (4.4–6.8)	14.2 (7.7–24.7)	6.5 (2.8–14.2)	52.5 (40.6–64.2)	26.8 (17.0–39.6)	
***Education (n=1870)***^b^						0.0022
≤High school	47.8 (45.3–50.4)	10.2 (8.3–12.6)	6.5 (4.4–9.7)	60.9 (56.9–64.7)*	22.4 (19.5–25.5)*	
≥Some college degree	52.2 (49.6–54.7)	13.8 (11.5–16.5)	7.5 (5.7–9.8)	50.0 (46.2–53.8)*	28.7 (25.5–32.1)*	
***Household income (n=1737)***						0.08
<$25,000	45.9 (43.0–48.8)	11.5 (9·2–14·3)	5.2 (3.6–7.7)	57.4 (53.5–61.2)	25.9 (22.8–29·3)	
$25,000 to $49,999	24.6 (22.8–26.5)	10.0 (7.6–13.2)	7.4 (5·0–11.0)	57.2 (51.6–62.6)	25.4 (20·7–30.7)	
≥$50,000	29.5 (26.6–32.5)	14.6 (11.5–18.3)	9·6 (6.5–14.0)	50.4 (44.5–56.4)	25.4 (20·6–30.8)	
***Employment status (n=1864)***						0.28
Full-time	45.8(43.0–48.7)	12.4 (10.1–15.1)	7.2 (5·2–9.7)	56.4 (52.4–60.4)	24.0 (20.5–27.9)	
Part-time	17.8 (15.8–19.9)	13.9 (10.3–18.5)	9.3 (6.2–13.6)	52.1 (46.2–57.9)	24.7 (20.7–29.3)	
Don’t currently work for pay	36.4 (33·3–39.6)	10.8 (8.5–13.6)	5.9 (4.1–8.5)	55.6 (51.6–59.5)	27.7 (24.5–31.2)	
***BMI, kg/m^2^(n=1823)***						0.27
<30·0	71.2 (68.8–73.4)	12.8 (10.9–15.0)	7.0 (5.4–9.1)	54.0 (50.9–57.1)	26.1 (23.4–29.0)	
≥30·0	28.8 (26.6–31.2)	9.4 (6.8–12·6)	7.7 (5.2–11.2)	58.1 (52.8–63.1)	24.8 (20.5–29.8)	
***US Census region (n=1870)***						0.46
Northeast	14.2 (12.3–16.3)	15.6 (11.5–20.8)	6.3 (3.5–11.1)	56.7 (50.9–62.4)	21.4 (16.3–27.6)	
Midwest	24.5 (22.4–26.6)	10.5 (8.8–12.5)	5.5 (3.4–9.0)	58.0 (54.2–61.8)	26.0 (22.1–30.2)	
South	40.2 (37·6–43·0)	11.6 (9.2–14.5)	7.2 (4.8–10.7)	54.2 (48.4–59.9)	27.0 (22.6–32.0)	
West	21.1 (18.5–23.9)	12.5 (9.4–16.4)	9.1 (6.3–13.0)	52.8 (47.4–58.1)	25.6 (20.6–31.3)	
**Behavioral factors**						
***Age at 1^st^ exposure to tobacco product (y) (n=1862)***						0.0016
<18	76.9 (74.8–78.9)	10.1 (8.4–12.1)*	6.7 (5.2–8.6)	56.5 (53.1–59.9)	26.7 (23.9–29.7)	
≥18	23.1 (21.1–25.2)	18.2 (14.2–22.9)*	8.5 (5.7–12.7)	51.2 (45.6–56.7)	22.2 (17.8–27.2)*	
***Duration of tobacco use (n=1869)***						0.001
Mean (SD)	24.2 (14.7)	20.0 (16.1)	24.3(15.9)	24.9 (14.1)	24.8 (14.6)	
***Other tobacco use (n=1870)***						0.006
Yes	25.6 (23.0–28.3)	16.8 (13.6–20.5)*	6.6 (4.5–9.6)	51.4 (46.8–56.0)*	25.2 (21.3–29.5)	
No	74.4 (71.7–77.0)	10.5 (9.0–12.2)*	7.2 (5·5–9.4)	56.5 (53.1–59.8)*	25.8 (23.1–28.8)	
***Alcohol use within 30 days (n=1616)***^c^						0.24
Yes	67.5 (64.0–70.9)	12.5 (10.4–14.9)	7.1 (5.4–9.4)	55.4 (51.7–59.0)	25.0 (22.0–28.2)	
No	32.5 (29.1–36.1)	10.3 (7.6–13.8)	6.6 (4.4–10.0)	53.2 (48.2–58.1)	29.9 (25.5–34.6)	
***Marijuana***^d^ ***(n=1245)***						0.09
Yes	31.8 (28.8–35.0)	15.4 (12.0–19.4)	6.6 (4.4–9.8)	53.7 (48.5–58.8)	24.3 (19.7–29.7)	
No	68.2 (65.1–71.2)	10.4 (8.3–13.1)	7.0 (5.0–9.7)	53.8 (49.6–57.9)	28.8 (25.2–32.8)	

Definitions: Cessation indicates no past-month tobacco use; harm reduction: e-cigarette mono use; cigarette transition: cigarette mono use. Abbreviations: CI indicates confidence intervals; US, United States, BMI, body mass index; SD, standard deviation.

a Other refers to Non-Hispanic American Indian or Alaska Native, Non-Hispanic Asian/Native Hawaiian or Other Pacific Islander and persons with multiple races.

b ≤ high school denotes less than high school/GED/high school graduate; ≥some college degree denotes some college/associate’s/bachelor’s/advanced degree.

c Answered by respondents who have ever used alcohol and used alcohol in the past 30 days.

d Answered by respondents who used marijuana in past 30days. Percentages are rounded up to 1 decimal place.

* indicates that those cell(s) are contributing to significant differences (or departure from null hypothesis of no association) between independent variable and 4-level transition outcome given that large residual errors between observed and expected cell frequencies add more to the overall chi-square statistic.

**Table 2 –:** Nicotine dependence symptoms, interest in quitting and clinical cardiovascular disease factors among adult (≥18 years) dual e-cigarette/cigarette according to main transitions at 2-year follow-up: Population Assessment of Tobacco and Health study, United States, 2013–2016

Tobacco use at 2-year follow-up (Wave 3)						
Wave 3 Wave 1	Overall %, (95% CI)	Cessation %, (95% CI)	Harm reduction %, (95% CI)	Cigarette transition %, (95% CI)	Dual use %, (95% CI)	P value
**Nicotine dependence**						
***Soon after waking, minutes (n=1621)***						0.98
<30	86.4 (84.3–88.1)	9.3 (7.9–10.9)	6.8 (5.2–8.9)	57.1 (53.5–60.5)	26.8 (23.9–29.9)	
≥30	13.6 (11.9–15.7)	8.7 (4.9–14.9)	7.6 (4.5–12.5)	57.3 (48.6–65.6)	26.4 (20.1–34.0)	
***Addicted to tobacco (n=1793)***						<0.0001
Yes	86.2 (84.0–88.2)	9.1 (7.7–10.7)*	6.6 (5.1–8.5)*	57.8 (54.5–61.1)*	26.5 (23.7–29.4)*	
No	13.8 (11.8–16.0)	26.6 (21.1–32.8)*	9.8 (6.4–14.7)*	42.2 (35.6–49.2)*	21.4 (16.0–28.0)*	
***Strong craving for tobacco product (n=1795)***						<0.0001
Yes	82.5 (80.5–84.3)	8.6 (7.2–10.4)*	6.4 (4.9–8.2)*	57.8 (54.1–61.4)*	27.2 (24.3–30.3)*	
No	17.5 (15.7–19.5)	25.0 (20.2–30.5)*	10.5 (6.8–15.9)*	45.6 (39.6–51.8)*	18.9 (14.6–24.0)*	
***Felt the need to use tobacco (n=1794)***						<0.0001
Yes	85.6 (83.5–87.4)	9.0 (7.6–10.7)*	6.9 (5.4–8.7)	57.5 (54.1–60.8)*	26.6 (23.8–29.7)	
No	14.4 (12.6–16.5)	26.0 (20.6–32.2)*	8.3 (5.2–13.1)	45.0 (37.9–52.3)*	20.7 (15.9–26.4)	
***Use tobacco in prohibited places (n=1795)***						0.04
Yes	27.9 (25.7–30.2)	7.9 (6.0–10.2)*	6.6 (4.4–9.8)	59.5 (54.4–64.5)	26.0 (22.0–30.6)	
No	72.1 (69.8–74.3)	12.9 (11.2–14.9)*	7.3 (5.5–9.6)	54.2 (50.7–57.6)	25.6 (22.8–28.7)	
***Gave up activities (n=1794)***						0.97
Yes	14.1 (12.5–15.8)	10.6 (7.4–14.9)	6.9 (3.8–12.3)	56.2 (49.4–62.8)	26.2 (20.3–33.2)	
No	85.9 (84.2–87.5)	11.6 (10.1–13.5)	7.1 (5.5–9.1)	55.6 (52.3–58.8)	25.7 (23.0–28.5)	
***Frequency of tobacco use (n=1631)***						<0.0001
Regular	64.1 (61.3–66.8)	6.2 (4.9–7.9)*	5.2 (3.7–7.3)*	63.8 (60.1–67.2)*	24.8 (22.0–28.3)
Not regular	35.9 (33.2–38.7)	15.2 (12.3–18.7)*	10.0 (7.4–13.4)*	45.0 (40.6–49.4)*	29.8 (26.0–33.9)
***ND symptoms severity categories (n=1796)***						<0.0001
0–3	22.1 (20.0–24.6)	25.3 (20.4–31.0)	9.6 (6.6–13.8)	45.1 (39.3–60.0)	20.0 (16.0–24.6)	
4–5	57.8 (55.2–60.4)	8.1 (6.4–10.3)*	7.5 (5.7–9.8)*	56.4 (52.7–60.1)	27.9 (24.7–31.4)	
6–7	20.1 (18.2–22.2)	6.0 (4.0–8.6)*	3.6 (2.1–6.2)*	65.0 (58.7–70.6)	25.6 (20.7–31.2)	
***Presence of ND symptoms (n=1796)***						<0.0001
Yes	96.7 (95.9–97.4)	10.2 (8.8–11.8)*	7.0 (5.5–8.9)*	56.6 (53.4–59.7)*	26.2 (23.7–28.9)*	
No	3.3 (2.6–4.1)	49.8 (35.7–64.0)*	13.5 (7.1–24.2)*	26.3 (15.7–40.5)*	10.5 (5.0–20.6)*	
***Interest in quitting scale (n=1280)***						0.70
1–3	11.9 (9.9–14.2)	11.2 (6.7–18.1)	4.8 (2.3–10.0)	59.8 (51.2–67.8)	24.2 (17.8–32.1)	
4–7	30.2 (27.6–33.0)	12.1 (9.0–16.1)	6.2 (3.7–10.2)	53.3 (47.3–59.1)	28.4 (23.4–34.1)	
8–10	57.9 (54.8–60.9)	10.0 (7.6–13.0)	7.6 (5.4–10.6)	54.1 (49.9–58.3)	28·3 (25.13–1.8)	
***Quit attempt (n=1007)***						0.32
≥1 (Yes)	83.3 (80.5–85.7)	11.1 (9.0–13.7)	7.9 (5.9–10.6)	52.5 (48.3–56.6)	28.5 (25.3–32.0)	
0 (No)	16.7 (14.2–19.5)	12.2 (7.4–19.5)	6.2 (3.1–12.1)	60.2 (50.2–69.5)	21.4 (14.5–30.3)	
***Presence of interest in quitting (n=1330)***						0.05
Yes	95.5 (94.1–96.5)	10.7 (8.9–12.9)	7.0 (5.3–9.1)	54.3 (50.9–57.7)	28.0 (25.2–31.0)	
No	4.5 (3.5–5.9)	23.2 (13.2–37.6)	6.9 (2.1–20.5)	53.9 (39.8–67.4)	16.0 (8.1–29.2)	
**Clinical CVD factors**						
***Diabetes (n=1866)***						0.92
Yes	10.6 (9.0–12.4)	11.3 (7.1–17.5)	8.2 (4.6–13.9)	54.1 (46.7–61.3)	26.4 (20.4–33.6)	
No	89.4 (87.6–91.0)	12.2 (10.6–14.0)	7.0 (5.4–8.9)	55.3 (52.3–58.2)	25.6 (23.1–28.2)	
***High cholesterol (n=1865)***						0.16
Yes	18.3 (16.4–20.4)	10.6 (7.4–14.9)	8.3 (5.2–13.2)	50.6 (44.2–57.1)	30.4 (25.1–36.4)	
No	81.7 (79.6–83.6)	12.4 (10.7–14.3)	6.7 (5.3–8.6)	56.2 (53.1–59.2)	24.7 (22.3–27.3)	
***Hypertension (n=1865)***						0.003
Yes	22.8 (20.4–25.4)	8.2 (5.8–11.7)*	5.6 (3.3–9.2)	63.9 (59.3–68.3)*	22.3 (18.3–26.8)	
No	77.2 (74.6–79.6)	13.2 (11.5–15.1)*	7.5 (5.8–9.6)	52.6 (49.3–55.9)*	26.8 (24.0–29.7)	
***Myocardial infarction (n=1865)***						0.88
Yes	3.2 (2.4–4.2)	8.8 (3.0–23.4)	6.2 (1.6–21.5)	55.3 (40.8–70.0)	29.7 (18.2–44.5)	
No	96.8 (95.8–97.6)	12.2 (10.6–13.9)	7.1 (5.6–8.9)	55.1 (52.3–58.0)	25.6 (23.2–28.2)	
**Presence of clinical CVD factors (n=1870)**						0.07
Yes	35.3 (32.4–38.3)	9.2 (7.0–11.9)	6.8 (4.7–9.7)	56.3 (51.8–60.6)	27.8 (23.8–32.1)	
No	64.7 (61.7–67.6)	13.5 (11.7–15.8)	7.2 (5.5–9.4)	54.6 (51.0–58.2)	24.5 (21.8–27.4)	

Definitions: Cessation indicates no past-month tobacco use; harm reduction: e-cigarette mono use; cigarette transition: cigarette mono use. Abbreviations: CI indicates confidence interval; CVD, cardiovascular disease; ND, nicotine dependence. Percentages are rounded up to 1 decimal place.

* indicates statistically significant differences between the transition categories. * indicates that those cell(s) are contributing to significant differences (or departure from null hypothesis of no association) between independent variable and 4-level transition outcome given that large residual errors between observed and expected cell frequencies add more to the overall chi-square statistic.

**Table 3 –:** Bivariate predictors of dual use transitions at 2-year follow-up among adults (≥18 years): Population Assessment of Tobacco and Health study, United States, 2013–2016

Predictors of dual use transitions at 2-year follow-up among adults (≥18 years)			
	Cessation	Harm reduction	Cigarette transition
	Unadjusted RRR (95% CI)	Unadjusted RRR (95% CI)	Unadjusted RRR (95% CI)
**Demographic and behavioral factors**			
*Age, years (ref: 18–24)*			
25–34	**0.54 (0.33–0.88)**	0.88 (0.49–1.53)	0.87 (0.60–1.26)
≥ 35	**0.41 (0**·**26–0.63)**	0.87 (0.50–1.50)	1.03 (0.74–1.43)
*Gender*			
Male (vs. female)	1.32 (0.95–1.83)	1.28 (0.87–1.89)	0.97 (0.78–1.21)
*Sexual orientation*			
Heterosexual (vs. lesbian/gay/bisexual/other)	1.68 (0.95–2.96)	1.06 (0.52–2.15)	1.32 (0.94–1.85)
*Race/ethnicity (ref: White)*			
Black	2.28 (1.11–4.68)	1.51 (0.52–4.38)	1.76 (1.00–3.12)
Hispanic	2.53 (1.60–4.02)	0.91 (0.43–1.95)	1.14 (0.82–1.59)
Other	1.41 (0.64–3.14)	0.90 (0.29–2.82)	0.97 (0.51–1.86)
*Education*^a^			
≥Some college degree (vs. ≤high school)	1.05 (0.76–1.46)	0.89 (0.53–1.51)	**0.64 (0.51–0.80)**
*Household income (ref: <$25,000)*			
$25,000–49,999	0.89 (0.57–1.39)	1.44 (0.77–2.68)	1.02 (0.72–1.44)
≥$50,000	1.30 (0.84–2.01)	1.86 (1.01–3.43)	0.90 (0.65–1.24)
*Employment (ref: full-time)*			
Part-time	1.09 (0.72–1.63)	1.26 (0.77–2.05)	0.90 (0.68–1.18)
Don’t work	0.75 (0.48–1.18)	0.72 (0.44–1.17)	0.85 (0.65–1.11)
*BMI*			
30 (vs. <30)	0.77 (0.50–1.19)	1.15 (0.69–1.91)	1.13 (0.85–1.50)
*US census region (ref: Northeast)*			
Midwest	**0.56 (0.33–0.94)**	0.73 (0.29–1.84)	0.85 (0.57–1.25)
South	0.59 (0.34–1.02)	0.90 (0.37–2.18)	0.76 (0.50–1.16)
West	0.67 (0.37–1.21)	1.22 (0.49–3.00)	0.78 (0.51–1.20)
*Age at 1^st^ exposure to tobacco product, years*			
<18 (vs. ≥18)	**0.46 (0.29–0.73)**	0.67 (0.39–1.13)	0.93 (0.67–1.31)
*Duration of tobacco use*	**0.98 (0.96–0.99)**	1.00 (0.98–1.01)	1.00 (0.99–1.01)
*Other tobacco use*			
Yes (vs. no)	**1.64 (1.18–2.29)**	0.94 (0.58–1.53)	0.93 (0.72–1.21)
*Past-month marijuana*			
Yes (vs. no)	**1.74 (1.14–2.65)**	1.12 (0.61–2.08)	1.18 (0.85–1.65)
*Past-month alcohol*			
Yes (vs.no)	1.45 (0.92–2.28)	1.29 (0.81–2.05)	1.25 (0.97–1.60)
**ND symptoms**			
*Soon after waking, minutes*			
<30 (vs. ≥30)	**1.06 (0.52–2.15)**	0.89 (0.45–1.78)	0.98 (0.65–1.49)
*Addicted to tobacco*			
Yes (vs. no)	**0.28 (0.17–0.45)**	**0.55 (0.31–0.97)**	1.11 (0.74–1.66)
*Strong craving for tobacco*			
Yes (vs. no)	**0.24 (0.15–0.38)**	**0.42 (0.25–0.70)**	0.88 (0.60–1.28)
*Felt the need to use tobacco*			
Yes (vs. no)	**0.27 (0.17–0.43)**	0.64 (0.37–1.12)	0.99 (0.66–1.48)
*Use tobacco in prohibited places*			
Yes (vs. no)	**0.60 (0.41–0.85)**	0.89 (0.51–1.56)	1.08 (0.83–1.40)
*Gave up activities*			
Yes (vs. no)	0.89 (0.53–1.49)	0.95 (0.44–2.04)	0.99 (0.70–1.41)
*Frequency of tobacco use*			
Regular (vs. not regular)	**0.49 (0.33–0.74)**	0.63 (0.38–1.03)	1.70 (1.33–2.18)
*ND symptoms severity (ref: 0–3)*			
4–5	**0.23 (0.14–0.38)**	**0.56 (0.35–0.90)**	0.89 (0.64–1.25)
6–7	**0.20 (0.11–0.31)**	**0.29 (0.14–0.61)**	1.12 (0.75–1.67)
*Interest in quitting scale (ref: 1–3)*			
4–5	0.92 (0.43–1.99)	1.10 (0.40–2.97)	0.76 (0.47–1.22)
6–7	0.76 (0.40–1.44)	1.35 (0.46–3.91)	0.77 (0.48–1.24)
*Quit attempt*			
Yes (vs. no)	0.68 (0.36–1.29)	0.95 (0.38–2.38)	0.65 (0.38–1.13)
**Clinical CVD factors**			
*Diabetes*			
Yes (vs.no)	0.90 (0.48–1.68)	1.13 (0.57–2.28)	0.95 (0.66–1.35)
*High cholesterol*			
Yes (vs.no)	0.69 (0.43–1.13)	1.00 (0.58–1.75)	**0.73 (0.55–0.98)**
*Hypertension*			
Yes (vs.no)	0.75 (0.45–1.25)	0.90 (0.49–1.66)	**1.46 (1.11–1.66)**
*Myocardial infarction*			
Yes (vs.no)	0.63 (0.14–2.88)	0.75 (0.14–4.02)	0.87 (0.44–1.72)
**ND symptoms domain**			
*Presence of ND symptoms*			
Yes (vs. no)	**0.08 (0.03–0.19)**	**0.21 (0.08–0.56)**	0.86 (0.34–2.19)
**Interest in quitting domain**			
*Presence of interest in quitting*			
Yes (vs. no)	**0.26 (0.10–0.74)**	0.58 (0.10–3.51)	0.58 (0.24–1.38)
**Clinical factors domain**			
*Presence of clinical CVD factors*			
Yes (vs. no)	**0.59 (0.40–0.88)**	0.84 (0.53–1.32)	0.91 (0.70–1.18)

Definitions: Cessation indicates no past-month tobacco use; harm reduction: e-cigarette mono use; cigarette transition: cigarette mono use.

Abbreviations: CI indicates confidence interval; CVD, cardiovascular disease; ND, nicotine dependence; RRR indicates relative risk ratio.

a ≤ high school denotes less than high school/GED/high school graduate; ≥some college degree denotes some college/associate’s/bachelor’s/advanced degree. Multinomial logistic regression modelling with dual use as reference group.

**Table 4 –:** Multivariate predictors of dual use transitions at 2-year follow up among adults (≥18 years): Population Assessment of Tobacco and Health study, United States, 2013–2016

Predictors of dual use transitions at 2-year follow-up among adults (>18 years)			
	Cessation	Harm reduction	Cigarette transition
	Adjusted RRR (95% CI)	Adjusted RRR (95% CI)	Adjusted RRR (95% CI)
**Demographic and behavioral factors**			
*Age, years (ref: 18—24)*			
25–34	0.74 (0.34–1.62)	1.33 (0.46–3.88)	0.90 (0.53–1.54)
≥ 35	0.37 (0.10–2.29)	1.38 (0.24–7.76)	1.23 (0.53–2.85)
*Gender*			
Male (vs. female)	1.21 (0.67–2.18)	1.19 (0.57–2.49)	0.96 (0.67–1.38)
*Race/ethnicity (ref: White)*			
Black	0.98 (0.23–4.05)	0.60 (0.10–6.60)	1.62 (0.62–4.22)
Hispanic	1.52 (0.64–3.62)	0.78 (0.20–3.04)	1.14 (0.67–1.96)
Other	1.65 (0.48–5.63)	0.21 (0.02–1.88)	1.21 (0.53–2.80)
*Education*^a^			
≥Some college degree (vs. ≤high school)	1·07 (0·59–1·94)	0.41 (0.17–1.00)	0.73 (0.50–1.05)
*Household income (ref: <$25,000)*			
$25,000–49,999	0.97 (0.48–1.92)	1.67 (0.62–4.51)	1.57 (0.96–2.55)
≥$50,000	1.19 (0.58–2.46)	2.71 (1.00–7.45)	1.16 (0.73–1.84)
*Age at 1^st^ exposure to tobacco product, years*			
<18 (vs. ≥18)	**0.44 (0.20–0.97)**	0.47 (0.16–1.44)	0.75 (0.41–1.38)
*Duration of tobacco use*	1.01 (0.95–1.06)	0.97 (0.92–1.02)	1·00 (0·99–1·01)
*Other tobacco use*			
Yes (vs. no)	1.52 (0.74–3.14)	0·75 (0.29–1.97)	0.88 (0.56–1.38)
*Past-month marijuana*			
Yes (vs. no)	1.02 (0.52–2.02)	0.68 (0.31–1.50)	0.98 (0.63–1.54)
*ND symptoms severity (ref: 0–3)*			
4–5	**0.38 (0.18–0.81)**	0.73 (0.30–1.76)	0.91 (0.57–1.46)
6–7	**0.29 (0.12–0.71)**	0.22 (0.04–1.08)	0.87 (0.50–1.50)
**ND symptoms domain**			
*Presence of ND symptoms*			
Yes (vs. no)	0.10 (0.01–1.02)	0.15 (0.01–3.21)	0.55 (0.03–10.28)
**Interest in quitting domain**			
*Presence of interest in quitting*			
Yes (vs. no)	1.30 (0.37–4.56)	0.98 (0.08–11.27)	0.79 (0.28–2.20)
**Clinical factors domain**			
*Presence of clinical factors*			
Yes (vs. no)	0.95 (0.44–2.05)	0.75 (0.30–1.87)	0.93 (0.56–1.54)

Definitions: Cessation indicates no past-month tobacco use; harm reduction: e-cigarette mono use; cigarette transition: cigarette mono use.

Abbreviations: CI indicates confidence interval; CVD, cardiovascular disease; ND, nicotine dependence; RRR, relative risk ratio.

a ≤ high school denotes less than high school/GED/high school graduate; ≥some college degree denotes some college/associate’s/bachelor’s/advanced degree. Multinomial logistic regression modelling with dual use as reference group. Only variables with p<0.2 from the bivariate associations were included in the final model. Model was adjusted for age, gender, race/ethnicity, education, income, age at first exposure to tobacco, duration of tobacco use, past-month marijuana, other tobacco use, marijuana, presence of ND symptoms, presence of interest in quitting and presence of clinical factors. Due to high correlation between ND severity and ND symptoms summary variables *(r=0.8, p<0.0001)*, we introduced them separately in the multivariate model.
